# Comparison of cancer prevalence between patients with glomerulonephritis and the general population at the time of kidney biopsy

**DOI:** 10.1371/journal.pone.0224024

**Published:** 2019-10-18

**Authors:** Jiwon Ryu, HyunJin Ryu, Sejoong Kim, Ho Jun Chin, Ki Young Na, Dong-Wan Chae, Hyung-Jin Yoon

**Affiliations:** 1 Department of Internal Medicine, Cheju Halla General Hospital, Cheju, Republic of Korea; 2 Department of Internal Medicine, Seoul National University College of Medicine, Seoul, Republic of Korea; 3 Department of Internal Medicine, Seoul National University Bundang Hospital, Seongnam, Republic of Korea; 4 Kidney Research Institute, Medical Research Center, Seoul National University College of Medicine, Seoul, Republic of Korea; 5 Department of Biomedical Engineering, Seoul National University College of Medicine, Seoul, Republic of Korea; University of KwaZulu-Natal, SOUTH AFRICA

## Abstract

Glomerulonephritis (GN) has been associated with many solid and hematologic malignancies. However, cancer prevalence at the time of GN diagnosis has been rarely examined. We aimed to evaluate the cancer prevalence in patients with GN at the time of kidney biopsy and to compare the results to those of the general population. A total of 1,155 patients who underwent kidney biopsy between 2003 and 2017 were included. We investigated patients diagnosed with cancer within one month of kidney biopsy. The occurrence of cancer was compared with that of the Korean general population using the observed-to-expected rates (O/E ratio). Twenty-nine patients with GN had cancer. The mean age of patients with and without cancer was 49 and 66 years old, respectively. The proportion of male patients with and without cancer was 49.4% and 58.6%, respectively. The glomerular filtration rate was different between the groups (78.1 ± 37.0, 58.0 ± 43.6 ml/min/1.73 m^2^, *p* = 0.006), but the urine protein/creatinine ratio was not (3.21 ± 4.01, 5.38 ± 7.47 g/gCr, *p* = 0.172). Immunoglobulin A nephropathy (IgAN) was the most common GN (37.9%), followed by membranous GN (13.5%), focal segmental glomerulosclerosis (9.7%), minimal change disease (9.2%), amyloidosis (1.2%). Amyloidosis was the most common GN associated with malignancy (20.7%). In patients with amyloidosis, cancer was observed almost 28 times more than expected and these patients showed higher cancer occurrence than patients with other GN (Relative Risk [RR]: 15.73; 95% confidence interval [CI]: 4.82–51.30; *p* < 0.01). Cancer occurrence was three times greater in GN patients aged > 50 years compared to the general population (O/E ratio: 3.42; 95% CI: 1.37–5.46; *p* = 0.027). Patients with GN, especially amyloidosis, have higher risk of cancer than the general population at the time of GN diagnosis. Older age (> 50 years) was one of the major determinants of the presence of cancer in GN patients.

## Introduction

Various solid and hematologic cancers have been associated with glomerulonephritis (GN). This phenomenon of GN and cancer has usually been thought to be a paraneoplastic syndrome, which is induced by the production hormones, cytokines, growth factors, and tumor antigens from tumor cells [1]. Most studies on the association between cancers and GN are focused on how GN is expressed in patients diagnosed with the diagnosis of cancer, what kinds of GN often develops in certain types of cancer, or cancer occurrence after the diagnosis of GN [2–4]. In some previous studies, there were cases in which cancer was diagnosed at the time of GN diagnosis [5–7]. There has not been much research into the concurrence of GN and cancer because of the tendency to think of GN as a paraneoplastic symptom of cancer. Moreover, it is difficult to assess the true prevalence of cancer in patients with GN because of some confounding factors [8]. First, there can be a detection bias (e.g., patients with membranous nephropathy are likely to be more aggressively screened for cancer). Second, demographic characteristics of the population such as age, sex, and smoking history, are variable. Therefore, cancer incidence at the time of GN diagnosis is rarely examined. Some studies have also evaluated the risk of cancer in GN patients versus the general population, but few studies have evaluated this risk of cancer at the time of GN diagnosis [9–11]. In this study, we aimed to investigate the prevalence of cancer at the time of GN diagnosis, in comparison to the general population using national data.

## Materials and methods

This retrospective study was approved by the Institutional Review Board (IRB) of Seoul National University Bundang Hospital (SNUBH) (No. B-1707/408-106). All patients were anonymized and identified by number. IRB waived the requirement for obtaining informed consents from the patients owing to the retrospective nature of data used.

### Study population

**Fig 1:**
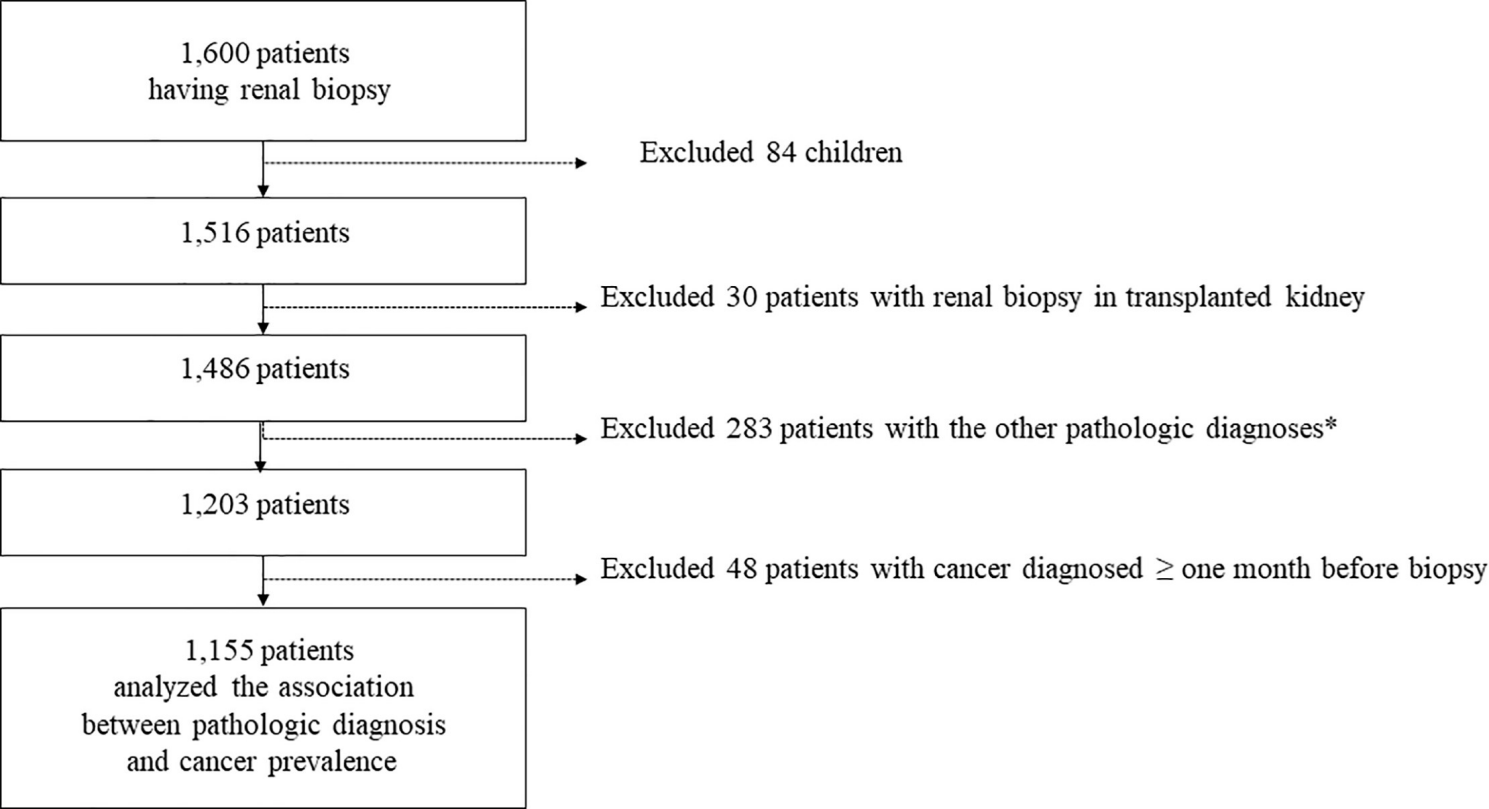
Selection of patients. *Pathologic diagnoses other than non-specific glomerulonephritis (GN), amyloidosis, crescentic GN, diabetic nephropathy, focal segmental glomerular sclerosis (FSGS), immunoglobulin (Ig)A nephropathy, lupus nephritis, minimal change lesion, membranous nephropathy, membranous proliferative GN (MPGN)-immune complex type, tubulointerstitial nephritis (TIN), or thrombotic microangiopathy (TMA).

From 2003 to 2017, 1,600 patients were diagnosed with GN through kidney biopsy at SNUBH. We confirmed the diagnoses these patients through electronic medical records database review. The criteria to perform kidney biopsy were as follows: consistent microscopic hematuria, urine protein-creatinine ratio (UPCR) of > 0.5 g/gCr, and renal dysfunction of unknown cause. Children (84 patients) and patients with transplanted kidney (30 patients) were excluded. Patients with a pathologic diagnosis (283 patients) other than the defined GN were excluded. Then 48 patients were further excluded because they were diagnosed with cancer more than one month prior to the kidney biopsy. Finally, a total of 1,155 patients were included. (Fig 1).

### Kidney pathology and clinical data

All the specimens were embedded in paraffin and stained with periodic acid-Schiff, Masson trichrome, methenamine silver, and haematoxylin-eosin for light microscopy. The immunofluorescence staining using antibodies against immunoglobulin (A, G, and M), complement (C) 3, C1q, kappa and lambda light chains, and electron microscopic (EM) examination were also performed. The pathologic diagnosis of kidney biopsies was confirmed by renal pathologist, and we have defined the GN for this study as non-specific GN, amyloidosis, crescentic GN (CresGN), diabetic nephropathy (DMN), focal segmental glomerular sclerosis (FSGS), IgA nephropathy (IgAN), lupus nephritis, minimal change disease (MCD), membranous nephropathy (MN), membranous proliferative GN (MPGN)-immune complex type, tubulointerstitial nephritis (TIN), or thrombotic microangiopathy (TMA). Baseline clinical data, including sex, age, anti-neutrophil cytoplasmic antibody (ANCA) specificity, and estimated glomerular filtration rate (eGFR) (determined by Chronic Kidney Disease Epidemiology Collaboration [CKD-EPI] equation), and UPCR, were obtained [12].

### Cancer diagnosis and incidence

The patients diagnosed with all types of cancer at SNUBH during the same period were reviewed, and we compared the date of initial cancer diagnosis with the date of kidney biopsy, then included the patients newly diagnosed within one month of the kidney biopsy. We evaluated what type of cancer occurred for each GN type.

### Cancer incidence in the general population

For the control group, we examined the cancer incidence in the general population. The National Cancer Center (NCC) registry contains data including ages and sexes, of those among the general Korean population who underwent health screening every 5-years. Among the patients registered in NCC, the data of the patients included in our GN group were collected. Moreover, the annual sex-specific incidence of cancer in the general population every 5-year interval was obtained from the National Statistical Office. With these data, we calculated the expected number of cancer incidence in our study as screening patients multiplied by the annual sex-specific cancer incidence of the general population. Then the actual observed cancer patients in the GN group was compared with the expected number of cancer incidence that is, the observed-to-expected ratio (O/E ratio). The NCC data was available only from 2003 to 2015; thus, we used the data of 978 patients in the GN group and compared their cancer incidence to that of the general population.

### Statistical analysis

Comparisons of continuous and categorical variables in the baseline characteristics were calculated using the Student 𝑡-test and chi-squared test, respectively. We evaluated the risk ratio of cancer incidence for each GN through a logistic regression analysis with adjustment for sex, age, laboratory data, and pathologic finding of biopsy. The cancer incidence in patients with GN were compared with that of the general population, and the 95% confidence interval (95% CI) for the O/E ratio was calculated. The O/E ratio in GN patients was analyzed by one sample *t*-test to evaluate whether the value was significantly greater or less than 1. A two-tailed *p* value of < 0.05 and 95% CI was considered statistically significant. We used the SPSS software, V. 19 (SPSS, Chicago, IL).

## Results

A total of 1,155 patients diagnosed with GN were included in the analysis. The mean age was 49.7 years, and 573 (49.6%) patients were male. The mean eGFR at the time of kidney biopsy was 77.6 ± 37.2 ml/min/1.73m^2^, and the mean UPCR was 3.26 ± 4.14 g/gCr. The proportion of patients with diabetes mellitus (DM) and hypertension (HTN) were 17.1% and 62.7%, repectively. The most diagnosed GN were as follows: IgAN (37.9%), MN (13.5%), FSGS (9.8%), MCD (9.3%), lupus nephritis (6.2%), DMN (5.3%), CresGN (4.8%), MPGN (3.8%), TIN (3.7%), non-specific GN (3.5%), amyloidosis (1.3%), and TMA (0.8%) (Table 1).

**Table 1:** Clinical characteristics of patients according to the presence of cancer at the time of kidney biopsy.

Characteristics*	Total (n = 1,155)	Cancer (-) (n = 1,126)	Cancer (+) (n = 29)	*p*-value^a^
**Age (year)** ^**b**^	49.7 ± 17.3	49.3 ± 17.2	66.4 ± 12.1	<0.001
**Sex (male, %)**	573 (49.6)	556 (49.4)	17 (58.6)	0.326
**History of CVD**	97 (8.4)	90 (8.0)	7 (24.1)	0.002
**History of CHD**	79 (6.8)	78 (6.9)	1 (3.4)	0.716
**DM**	197 (17.1)	193 (17.5)	4 (13.8)	0.805
**Hypertension**	724 (62.7)	706 (62.7)	18 (62.1)	0.464
**SBP (mmHg)** ^**b**^	129.1 ± 19.4	129.1 ± 19.3	129.8 ± 23.0	0.911
**DBP (mmHg)** ^**b**^	73.7 ± 12.1	73.7 ± 12.1	71.1 ± 11.7	0.305
**Protein (g/dl)** ^**b**^	6.1 ± 1.1	6.1 ± 1.1	5.7 ± 1.2	0.041
**Albumin (g/dl)** ^**b**^	3.4 ± 0.8	3.4 ± 0.8	2.8 ± 0.9	0.001
**Bilirubin (mg/dl)** ^**b**^	0.50 ± 0.37	0.50 ± 0.37	0.45 ± 0.19	0.717
**Cholesterol (mg/dl)** ^**b**^	214 ± 87	214 ± 87	199 ± 91	0.109
**Glucose (mg/dl)** ^**b**^	118 ± 45	118 ± 45	106 ± 29	0.064
**Hemoglobin (g/dl)** ^**b**^	12.5 ± 2.3	12.5 ± 2.3	11.1 ± 2.1	0.001
**Creatinine (mg/dl)** ^**b**^	1.43 ± 1.63	1.40 ± 1.59	2.40 ± 2.52	0.048
**GFR (ml/min/1.73 m**^**2**^**)** ^**b**^	77.6 ± 37.2	78.1 ± 37.0	58.0 ± 43.6	0.006
**UPCR (g/gCr)**^**b**^	3.26 ± 4.14	3.21 ± 4.01	5.38 ± 7.47	0.172
**Albumin by dipstick (≥2+)**	840 (72.7)	820 (76.1)	20 (74.1)	0.804
**Pathologic diagnosis**				<0.001
Non-specific GN	41 (3.5)	40 (3.6)	1 (3.4)	
Amyloidosis	15 (1.3)	9 (0.8)	6 (20.7)	
Crescentic GN	56 (4.8)	50 (4.4)	6 (20.7)	
Diabetic nephropathy	61 (5.3)	60 (5.3)	1 (3.4)	
FSGS	113 (9.8)	110 (9.8)	3 (10.3)	
IgA nephropathy	438 (37.9)	435 (38.6)	3 (10.3)	
Lupus nephritis	72 (6.2)	71 (6.3)	1 (3.4)	
MCD	107 (9.3)	104 (9.2)	3 (10.3)	
MN	156 (13.5)	155 (13.8)	1 (3.4)	
IC type of MPGN	44 (3.8)	43 (3.8)	1 (3.4)	
TIN	43 (3.7)	40 (3.6)	3 (10.3)	
TMA	9 (0.8)	9 (0.8)	0 (0.0)	

*CVD: cerebrovascular disease, CHD: coronary heart disease, DM: diabetes mellitus, SBP: systolic blood pressure, DBP: diastolic blood pressure, eGFR: estimated glomerular filtration rate (eGFR) (determined by Chronic Kidney Disease Epidemiology Collaboration (CKD-EPI) equation), UPCR: urine protein/creatinine ratio, GN: glomerulonephritis, FSGS: focal segmental glomerulosclerosis, MCD: minimal change disease, MN: membranous nephropathy, IC type of MPGN: immune complex type of membranoproliferative GN, TIN: tubulointerstitial nephritis, TMA: thrombotic microangiopathy

^a^Difference between the initial and follow-up groups by using paired *t*-test

^**b**^Continuous variables expressed as mean ± standard deviation

### Observed cancer in GN patients

At the time of kidney biopsy, 29 patients had cancer (Table 2). Amyloidosis (20.7%, 6/29) and CresGN (20.7%, 6/29) were the GN types most associated with cancer. The cancer associated with amyloidosis was multiple myeloma (MM) (n = 6). CresGN was associated with lung cancer (2), colon cancer (1), Kaposi’s sarcoma (1), MM (1), and stomach cancer (1). In IgAN, which is the most frequent GN type, 3 cancer patients (10.7%) were diagnosed at the time of kidney biopsy, with colon cancer in 1 patient, metastatic tumor to the spine in another, and thyroid cancer in the last. Three patients (10.7%) with FSGS, MCD, and TIN had cancer. In MN, which is known to be most associated with cancer, only one cancer patient was diagnosed with cancer (breast cancer). Overall, MM was the most frequently diagnosed cancer type in patients with GN at the time of kidney biopsy, followed by other common cancer types such as stomach cancer (4), colon cancer (2), thyroid cancer (2), lymphoma (2), and lung cancer (2).

**Table 2:** The types of cancer diagnosed at the time of kidney biopsy.

GN*	Cancer (n)
**Non-spf** **(41)**	Malignant neoplasm of abdomen (1)
**Amyloidosis (15)**	Multiple myeloma (6)
**Crescentic GN (56)**	Colon cancer (1) Kaposi's sarcoma (1) Lung cancer (2) Multiple myeloma (1) Stomach cancer (1)
**DMN** **(61)**	Thyroid cancer (1)
**FSGS** **(113)**	Multiple myeloma (1) Renal cell carcinoma (1) Transitional cell carcinoma (1)
**IgAN** **(438)**	Colon cancer (1) Metastatic tumor to the spine (1) Thyroid cancer (1)
**LN** **(72)**	Lymphoma (1)
**MCD** **(103)**	Epithelial ovarian cancer (1) Stomach cancer (2)
**MN** **(156)**	Breast cancer (1)
**MPGN** **(44)**	Lymphoma (1)
**TIN** **(43)**	Gallbladder cancer (1) Multiple myeloma (1) Stomach cancer (1)
**TMA** **(9)**	0
Total GN patients (1155)	Total cancer patients (29)

* GN: glomerulonephritis, Non-spf: non-specific GN, Amyl: amyloidosis, CresGN: crescentic GN, DMN: diabetic nephropathy, FSGS: focal segmental glomerulosclerosis, IgAN: IgA nephropathy, LN: lupus nephritis, MCD: minimal change disease, MN: membranous nephropathy, IC type of MPGN: immune complex type of membranoproliferative GN, TIN: tubulointerstitial nephritis, TMA: thrombotic microangiopathy

Regarding the evaluation of cancer risk for each GN in patients with and without GN (Table 3), amyloidosis showed a high risk of cancer in univariate analysis (Relative Risk [RR]: 32.3; *p* < 0.01). In the additional analyses with further adjustments for age, sex, history of CVD, serum albumin, hemoglobin, eGFR, and pathologic findings of GN, the risk of cancer was 15.7 (95% CI: 4.82–51.30; *p* < 0.01). In the RR for cancer of crescentic GN, the risk was significantly high in the univariate analysis, but not in the multivariate analysis (RR: 5.61; 95% CI: 2.18–14.40; *p* < 0.001).

**Table 3:** The risk of cancer incidence according to the pathologic diagnosis of kidney biopsy.

	Model 1^a^			Model 2^b^			Model 3^c^		
	RR^d^	95% CI^e^	*p*	RR	95% CI	*p*	RR	95% CI	*p*
**Amyloidosis**	32.37	10.64–98.48	<0.001	19.28	6.088–61.08	<0.001	15.73	4.825–51.30	<0.001
**Crescentic GN**^f^	5.61	2.188–14.403	<0.001	NA^g^	NA	NA	NA	NA	NA
**IgA nephropathy**	0.18	0.055–0.609	<0.001	NA	NA	NA	NA	NA	NA

^a^Model 1: univariate logistic regression

^b^Model 2: adjusted for age and sex

^c^Model 3: adjusted for age, sex, factors of incident cancer, such as history of CVD, and serum level of hemoglobin, albumin, eGFR, and pathologic findings such as cellularity in glomeruli, degree of glomerular segmental sclerosis, and intensity of complement 3 deposition in glomeruli

^d^RR: relative risk

^e^95% CI: 95% confidence interval

^f^Crescentic glomerulonephritis

^g^NA: not analyzed because of the small number of patients

### Comparison of patients with or without cancer

The clinical characteristics of the groups according to cancer type are presented in Table 2. Significant differences between groups were found with respect to age, history of cerebrovascular disease, serum protein, serum albumin level, hemoglobin, serum creatinine and eGFR. Cancer patients were older (mean age: 66.4 vs 49.3 years) and had CVD more frequently than did non-cancer patients (24.1% vs 8.0%). DM and HTN were not different between the patients with cancer and without cancer. Serum protein, albumin, hemoglobin, and eGFR levels were lower in cancer patients than in non-cancer patients.

### Comparison of cancer incidence between GN patients and the general population

In our GN patients, the risk of cancer in all GN types was significantly high. The O/E ratio of overall cancer was 2.45 (95% CI: 1.00–3.89). In age-stratified analyses, the O/E ratio showed a high value in elderly patients aged > 50 years (O/E ratio: 3.42; 95% CI: 1.37–5.46). In the age and sex-stratified analyses, the ratio was consistently high in all patients aged > 50 years, regardless of sex, whereas patients ≤50 years showed a ratio of <1. Moreover, the O/E ratio in patients aged ≥50 years was significant (*p* = 0.027), especially in the elderly female patients (O/E ratio: 4.61; 95% CI: 1.49–7.73; *p* = 0.029) (Table 4).

**Table 4:** Observed, expected, and observed/expected ratio of cancer at the time of kidney biopsy from 2003 to 2015.

		Number^a^	Observed^b^	Expected^c^	O/E^d^ (95% CI)^e^	*p*^f^
**All**	**Total**	978	23	6.15	2.45 (1.00–3.89)	0.050
	**Age < 50 years**	503	3	0.96	1.33 (-0.97–3.64)	0.753
	**Age ≥ 50 years**	475	20	5.19	3.42 (1.37–5.46)	0.027
**Male**	**Total**	484	12	4.02	1.75 (0.23 -– 3.27)	0.307
	**Age < 50 years**	241	1	0.27	1.21 (-1.74–4.15)	0.870
	**Age ≥ 50 years**	243	11	3.74	2.28 (0.22–4.23)	0.191
**Female**	**Total**	494	11	2.60	2.93 (0.90–4.97)	0.061
	**Age < 50 years**	262	2	0.75	1.01 (-1.46–3.48)	0.993
	**Age ≥ 50 years**	232	9	1.85	4.61 (1.49–7.73)	0.029

^**a**^Patients with GN who matched the data of the National Cancer Center from 2003 to 2015

^**b**^ Cancer cases occurred in patients with GN

^**c**^Expected number: calculated as the number of screened patients in our study multiplied by corresponding sex-specific cancer incidence of the general population by year from the National Statistical Office

^**d**^O/E ratio: number of observed patients divided by expected number

^**e**^95% CI: 95% confidence interval

^**f**^One sample t-test analysis was performed to evaluate if the value was significantly greater or less than 1

### Comparison of cancer incidence between each GN type and the general population

The cancer risk of each GN type was analyzed against the general population using O/E ratio (Table 5). In most of the GNs, except for MN, the risk of cancer was higher than that of the general population. Amyloidosis, although not statistically significant, showed a higher risk of cancer than any other GN types (O/E ratio: 28.57; *p* = 0.236). In sequence, the incidence of cancer was higher in patients with TIN than in the general population (O/E ratio: 10.21; *p* = 0.15).

**Table 5:** Observed, expected, and observed/expected ratio of cancer in each glomerulonephritis (GN) from 2003 to 2015.

GN^a^	Number^b^	Observed^c^	Expected^d^	O/E^e^(95% CI)^f^	*p*^g^
**Non-specific GN**	39	1	0.24	4.23 (-11.3–30.2)	0.39
**Amyloidosis**	14	5	0.18	28.57 (-24.6–83.3)	0.236
**Crescentic GN**	45	3	0.54	5.55 (-0.68–6.31)	0.271
**Diabetic nephropathy**	55	1	0.45	2.25 (-2.22–6.01)	0.646
**FSGS**	97	2	0.63	3.15 (-1.15–4.41)	0.636
**IgA nephropathy**	369	3	1.50	2.01 (-0.41–3.43)	0.578
**Lupus nephritis**	60	1	0.11	9.33 (-5.65–14.3)	0.464
**MCD**	89	2	0.57	3.48 (-1.82–9.71)	0.292
**MN**	131	1	1.23	0.81 (-0.43–1.20)	0.13
**IC type of MPGN**	38	1	0.34	2.93 (-0.81–2.20)	0.666
**TIN**	32	3	0.29	10.21 (-1.33–14.0)	0.15
**TMA**	9	0	0.07	NA ^h^	NA

^a^FSGS: focal segmental glomerulosclerosis, MCD: minimal change disease, MN: membranous nephropathy, IC type of MPGN: immune complex type of membranoproliferative GN, TIN: tubulointerstitial nephritis, TMA: thrombotic microangiopathy

^b^Patients with GN who were matched to the data of National Cancer Center from 2003 to 2015

^**c**^Cancer cases occurring in patients with GN

^**d**^Expected number: was calculated as the number of screened patients in our study multiplied by corresponding sex-specific cancer incidence of general population by year from the National Statistical Office

^**e**^O/E ratio: observed patients number divided by expected number

^**f**^95% CI: 95% confidence interval

^**g**^One sample t-test analysis was performed to evaluate if the value was significantly greater or less than 1

^h^NA: not analyzed because of the small number of patients

## Discussion

This study aimed to evaluate the cancer prevalence in patients with GN at the time of kidney biopsy and to compare the results to those of the general population. Several studies on cancer and GN have mostly evaluated GN as a paraneoplastic syndrome after cancer occurrence or, conversely, have evaluated the prevalence of cancer over the years following GN diagnosis [1, 3, 9, 11–17]. However, in some studies, cancers were diagnosed at the time of GN diagnosis [5–8]. Therefore, we evaluated the incidence and risk of cancer at the time of GN diagnosis excluding other influences. Moreover, we tried to avoid multiple biases (such as diagnoses in different departments, ambiguous symptoms or criteria of diagnosis, among others) through a population-based approach.

Our study showed the various cancer types occurring in patients with GN at the time of diagnosis. MM occurred in all patients with amyloidosis. The association of amyloidosis with MM has been well known. Ig amyloidosis represents a more nephrotic feature with preserved renal function than the other types of amyloidosis, which leads to their early diagnosis [18, 19]. Therefore, when MM and AL amyloidosis coexist, the myeloma could be diagnosed before or around the time of the amyloidosis diagnosis [20]. One recent study showed that the risk of MM was the highest in the period of one year after kidney biopsy in patients with GN [17]. CresGN was diagnosed simultaneously with several malignancies (colon cancer, lung cancer, stomach cancer, Kaposi's sarcoma, and MM), which are the same as those known to be associated with CresGN [2, 6, 16, 21, 22]. As in this study, there were cases in which the cancer diagnosis was established around the discovery of CresGN. In one case, hidden gastric cancer was found through an autopsy of a patient who died 2 months after the diagnosis of CresGN [22–26]. There have been reports of cancers in patients with FSGS, IgAN, and TIN, with cancer and these GNs being diagnosed almost simultaneously [2, 16, 27, 28]. In our patients with MCD, only solid tumors (ovarian and stomach cancer) occurred. MCD is known to be associated with lymphoma and leukemia, and its association with solid tumors has been clarified [1, 5]. There were also some cases of solid tumors diagnosed in the work-up process soon after the diagnosis of MCD [29, 30].

The most surprising finding in this study is that the occurrence of cancer in MN was very low. The cancer diagnosed at the time of MN diagnosis in our study was only one case (breast cancer). Various studies reported considerable cases wherein MN and cancer were simultaneously diagnosed, and we expected the patients with MN to have more cancer occurrence [2, 8, 14, 31–33]. However, our result was different from the findings of previous studies, which may be due to the small number of total cancer patients.

Many studies have suggested pathological hypotheses about the association between cancer and GN [1, 19, 28, 30, 34, 35]. They proposed etiological mechanisms including tumor-antibody production against tumor antigens, circulating factor secreted by T lymphocytes, B-cell production of cryoglobulin, and M-component and circulating Ig-A. Because of the immune perturbations in cancer patients, antibodies to exogenous or endogenous antigens could lead to the occurrence of immune-complex nephritis in vulnerable patients. It is unknown how these immunological mechanisms affected the time-interval between the occurrence of cancer and GN. Moreover, there are arguments that the cancer that is first manifested as a nephrotic syndrome has a grave course and poor prognosis [6, 14]. In the previous studies, the researchers recommended that the cancer should be suspected and evaluated in patients diagnosed with GN because GN may be evidence of silent cancer. In practice, the KDIGO guidelines recommend malignancy screening only for MN, FSGS, and IgAN, among other GNs.

Almost all previous studies so far showed that the risk of cancer is higher in elderly patients with GN, especially those aged > 60 years. In our study, the age-based criterion for increasing cancer incidence was 50 years old. Especially, the incidence of cancer in women aged > 50 years was significantly higher than that in men. In terms of sex-based differences, the results showed a slightly different trend between studies.

This study has several limitations. First, this work was a single-center study, and the GN and cancer types seemed to be not variable. Second, some clinical information was unavailable, such as the presence of HIV and hepatitis B and C, which are related to immune tolerance. Third, we did not mention whether there were any differences in pathologic findings between the GNs in cancer patients, because there were no significant differences according to the pathologic findings. However, one of the strengths of this study was its population-based approach. Comparing the risk of cancer occurrence with the average value of the general population can be a good indication of the degree of risk. Moreover, this study is unique as it assesses the occurrence of cancer only at the time of GN diagnosis.

## Conclusion

We have demonstrated that 29 GN patients were diagnosed with cancer at time of kidney biopsy. Amyloidosis presented to be the most common GN associated with cancer occurrence and showed a higher risk of cancer compared to the general population. In all patients with GN, the risk of cancer was definitely higher than that of the general population, especially those aged ≥ 50 years. Thus, cancer screening for patients aged ≥ 50 years who are diagnosed with GN is required at the time of GN diagnosis.
